# A High-Throughput DNA Sequence Aligner for Microbial Ecology Studies

**DOI:** 10.1371/journal.pone.0008230

**Published:** 2009-12-14

**Authors:** Patrick D. Schloss

**Affiliations:** 1 Department of Microbiology, University of Massachusetts, Amherst, Massachusetts, United States of America; 2 Department of Microbiology and Immunology, University of Michigan, Ann Arbor, Michigan, United States of America; Dana-Farber Cancer Institute, United States of America

## Abstract

As the scope of microbial surveys expands with the parallel growth in sequencing capacity, a significant bottleneck in data analysis is the ability to generate a biologically meaningful multiple sequence alignment. The most commonly used aligners have varying alignment quality and speed, tend to depend on a specific reference alignment, or lack a complete description of the underlying algorithm. The purpose of this study was to create and validate an aligner with the goal of quickly generating a high quality alignment and having the flexibility to use any reference alignment. Using the simple nearest alignment space termination algorithm, the resulting aligner operates in linear time, requires a small memory footprint, and generates a high quality alignment. In addition, the alignments generated for variable regions were of as high a quality as the alignment of full-length sequences. As implemented, the method was able to align 18 full-length 16S rRNA gene sequences and 58 V2 region sequences per second to the 50,000-column SILVA reference alignment. Most importantly, the resulting alignments were of a quality equal to SILVA-generated alignments. The aligner described in this study will enable scientists to rapidly generate robust multiple sequences alignments that are implicitly based upon the predicted secondary structure of the 16S rRNA molecule. Furthermore, because the implementation is not connected to a specific database it is easy to generalize the method to reference alignments for any DNA sequence.

## Introduction

Recent advances in traditional Sanger sequencing and pyrosequencing technologies have facilitated the ability to design studies where 10^2^−10^7^ 16S rRNA gene sequences ranging in length between 60 and 1500 bp are generated to address interesting ecological questions [1]–[4]. This data gush has forced computational microbial ecologists to re-factor software tools to make the analysis of these datasets feasible. A significant bottleneck in the analysis of these sequences is the generation of a robust multiple sequence alignment (MSA). An MSA is critical to generating phylogenies and calculating meaningful pairwise genetic distances that can be used to assign sequences to operationally-defined taxonomic units [OTUs, 5]. Because of the difficulty inherent in MSA calculations, investigators have bypassed OTU-based approaches in preference for phylotype-based approaches [3], [6]. In such approaches, sequences are assigned to bins based on similarity to a curated database. This has the limitation that sequences in the same phylotype may be only marginally similar to each other or unknown sequences may not affiliate to a pre-existing taxonomy. Therefore, there is a significant need to reassess alignment techniques with regard to their speed, memory requirements, and accuracy.

For generic sequencing alignments, popular aligners have included ClustalW [7], MAFFT [8], and MUSCLE [9]. Several recent pyrosequencing studies of the V6 16S rRNA region (ca. 60 bp long) have used MUSCLE to generate MSAs for up to 20,000 sequences [3], [10], [11]. These techniques scale at least quadratically in space and time for sequence length and quadratically in space and to the third power in time for the number of sequences. Thus, as the number of sequences in a dataset surpasses their length, the memory required to double the number of sequences in an alignment increases at least four-fold and the time required increases at least eight-fold. Because these limitations are compounded in typical implementations by storing all of the data in RAM, it is not possible to align more than 5,000 full-length sequences on a typical desktop computer. Alternatively, some have proposed calculating genetic distances using only pairwise alignments [12]. The time requirements of such an approach scale quadratically with the number of sequences and makes it impossible to insure positional homology. An additional limitation of the generic sequence aligners is that the alignments do not incorporate the predicted secondary structure of the 16S rRNA molecule and therefore it is impossible to compare datasets without re-aligning all of the sequences.

The secondary structure is an important feature to consider in generating the alignment because it increases the likelihood that the alignment conserves positional homology between sequences [13]. Without such a consideration, the alignment is more sensitive to user-supplied parameters such as match and mismatch scores, and gap opening and extension penalties. There are currently four profile-based aligners that are used to generate 16S rRNA-specific alignments that each at least implicitly considers the secondary structure. Each of these methods is associated with well-established 16S rRNA gene databases and reference MSAs, which each have strengths and weaknesses. A general advantage of each of these methods is that rather than generating alignments de novo, they perform profile-based alignments and their complexity scales linearly in time and have a minimal memory footprint. In deciding upon an aligner it is important to consider the alignment quality, ability to align large datasets, speed, dependence on a specific database, cost, and openness of the algorithm.

The RDP (http://rdp.cme.msu.edu) aligner uses 16S rRNA secondary structure models to generate and apply hidden Markov models within Infernal [14], [15]. Although this has the strength of directly incorporating the secondary structure of the 16S rRNA molecule, the number of available models is limited, which causes variable regions to not be aligned. Furthermore, while the aligner scales linearly in time (i.e. doubling the number of sequences doubles the time required to construct the MSA), the alignment process is relatively slow compared to other methods. Finally, the length and structure of the RDP alignment changes as new reference models are included. This requires users to re-align their data each time they acquire new sequences. The strengths of the RDP aligner are that it is free, open source, and can be run on a user's local computer.

The popular software package, ARB [16], has a built-in aligner that has yet to be fully described in the literature. Although specific details are lacking, the aligner uses a suffix tree to find related reference sequences and that the actual alignment step uses multiple reference sequences and secondary structure information to carryout the alignment. Perhaps the most significant limitation in the ARB implementation is that suffix tree server has become practically unusable to most users as the number of full-length sequences has increased. As an alternative, the ARB developers have spun-off the SILVA database project (http://www.arb-silva.de). SILVA serves as a repository for aligned rRNA sequences and the SINA aligner [17]. This implementation of the ARB aligner is more convenient, but also has yet to be described in the literature. Complicating matters is that the website limits users to aligning 300 sequences at a time; aligning additional sequences is available on a pay-for-use basis. Although the MSA length and structure is stable, it is an unwieldy 50,000 columns long.

**Figure 1:**
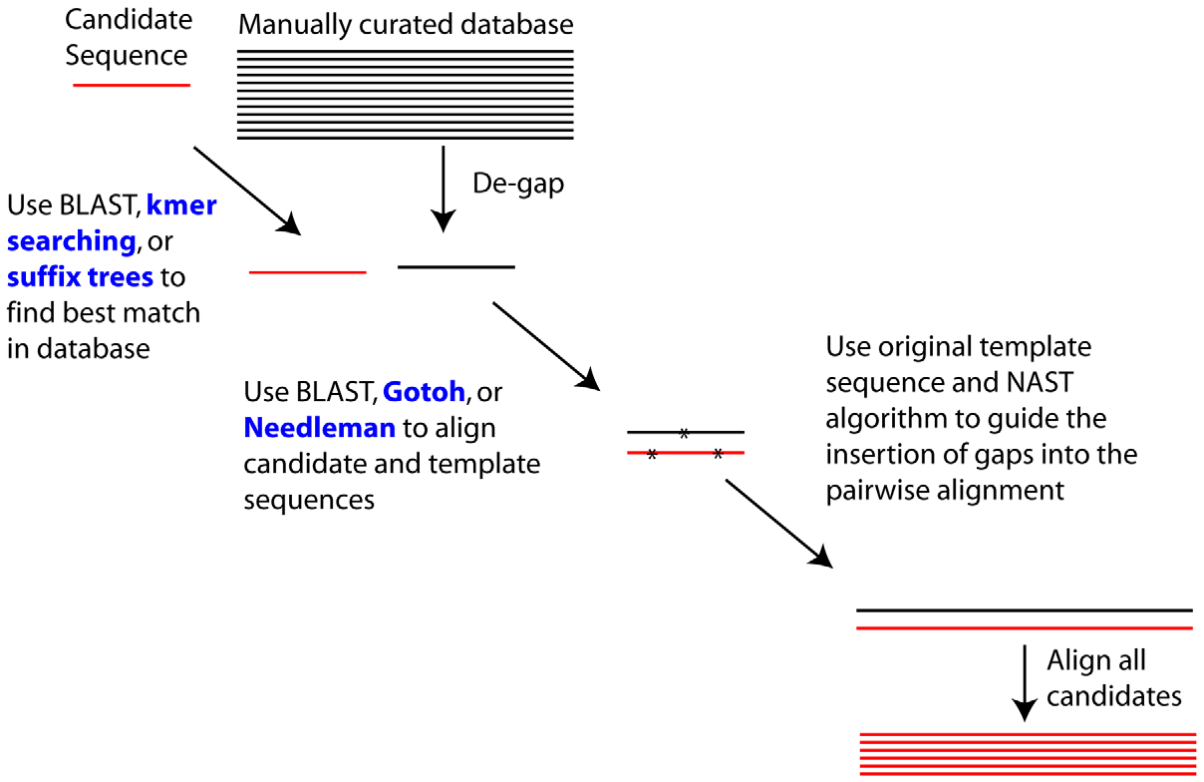
Flowchart describing the alignment algorithm. The published and current greengenes aligner algorithm is shown in black and the modifications that were tested in this study are shown in blue.

The aligner available through the greengenes website (http://greengenes.lbl.gov) is not explicitly dependent on secondary structure models; however, the generation of the reference database alignment does take into account the secondary structure (Fig. 1) [18], [19]. The reference alignment is considerably shorter than the SILVA alignment (7,682 columns). Although the source code for the greengenes aligner is not open, the algorithm has been published [19]. The original implementation used kmer searching with 7-mers to identify the closest template sequence in the reference database. The current implementation uses blastn [20] to identify the longest template sequence among the top-ten matches (TZ DeSantis, personal communication). In the second step, the algorithm uses blastn to generate pairwise alignments between the unaligned candidate and template sequences; in the current greengenes implementation the same blastn alignment generated in the first step is used for the second step. Finally, gap positions are introduced to the candidate sequence so that the final alignment is the same length as the reference database and positional homology is maintained using the nearest alignment space termination (NAST) algorithm. The speed of the aligner scales linearly with the number of candidate sequences so that doubling the number of candidate sequences would double the time requirement. The only significant memory requirement is what is required to store the reference alignment. A challenge in each of these profile-based methods is the creation of a high-quality reference alignment because the alignments that are generated will only be as good as the reference. Assuming that one has a good reference alignment, the greengenes aligner appears to have the most potential for quickly generating a high quality sequence alignment.

In the present study I used several simulations to assess how various permutations of the alignment algorithm effect alignment quality and speed. Specifically, I tested various methods of identifying the best template sequence and completing the pairwise alignments (Fig. 1). I was also interested in determining how well these results generalized to various regions within the 16S rRNA gene sequence commonly used in recently published surveys. This study enabled me to produce an aligner that rapidly produced high-quality alignments, robust to analysis of sequence fragments, could be generalized to genes other than 16S rRNA, and made independent of a specific database.

## Results

### Comparison of Database Quality

**Figure 2:**
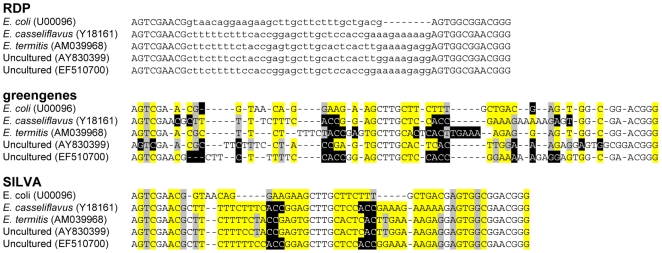
Comparison of alignments generated by the RDP, greengenes, and SILVA databases. Alignments were taken between positions 60 and 113 of the E. coli 16S rRNA gene sequence for *E. coli* and four *Enteroccocus* spp. The alignment generated for these sequences within this region using 8-mers and the Needleman-Wunsch algorithm was identical to that found in the SILVA alignment. The lower-case bases in the RDP alignment indicate unaligned positions. For the greengenes and SILVA alignments, yellow-highlighting represents bases that are predicted to form traditional Watson-Crick base-pairs in the secondary structure, gray-highlighting represents weak base-pairs, black-highlighting represents bases that will not form base-pairs, and a lack of highlighting represents bases that are predicted to be in loop structures.

I identified 200,433 unique, high-quality, aligned 16S rRNA gene sequences that were shared by the SILVA, greengenes, and RDP databases. ARB databases provided by SILVA and greengenes include helical mapping data that indicate which bases pair with each other within the predicted 16S rRNA secondary structure. For example, using the SILVA alignment, the 1,542 bp *E. coli* 16S rRNA secondary structure consists of 1,028 bases that are expected to form pairs. Of these, 808 form normal Watson-Crick base pairs (i.e. AT, GC; marked with a ‘∼’ in ARB), 202 form weak pairs (i.e. GA, TT, GT; marked ‘−’, ‘ = ’, or ‘+’), and 18 do not pair (i.e. AA, AC, CC, CT, GG; marked ‘#’). Although sequences naturally have non-pairing bases within the paring regions of the secondary structure, an excessive number of these is an indicator of poor sequence alignment (e.g. Fig. 2). With this in mind, I compared the number of non-paring bases in each sequence from the SILVA and greengenes databases. On average, each sequence alignment had 18.0 more non-paring bases (sd = 16.0) in the greengenes alignment than in the SILVA alignment; only 5.8% of the greengenes aligned sequences had fewer non-pairs than the SILVA aligned sequences. It was not possible to perform a similar analysis with the RDP alignment because similar helical mapping data are not available. Rather, I counted the number of unaligned bases in each sequence, which they indicate in a lower case typeface to characterize the RDP alignment (Fig. 2). On average, 7.6% of each sequence's bases were unaligned (sd = 1.5%) and within the variable regions targeted by pyrosequencing the percentage was higher. Based on these analyses, I decided to use the SILVA MSA to evaluate the new aligner.

### Database Searching Methods

The first step in the alignment algorithm is to find the most similar template sequence for each unaligned candidate sequence. The ARB aligner uses a suffix tree search method and the greengenes aligner has used either a kmer searching method or blastn. Suffix tree and kmer searching have the advantage that they are alignment independent techniques, which could make them considerably faster than using the alignment-based blastn approach. The extent of the speed-up and the effect on accuracy were unknown. To assess the accuracy, I calculated the similarity between each SILVA-aligned candidate sequence and each of the template sequences. These similarities were used to assess how well each method identified the best match and resulting alignment quality.

Regardless of the region within the 16S rRNA gene, kmer searching outperformed both blastn and suffix tree searching in its ability to find the best template (Tables 1 and S1). When searching against full-length templates, 7 to 9-mers provided the closest matches. Across the 10 regions that I tested, between 68 and 77% of the candidates found their true best template match. With the exception of the V6 region, the average candidate sequence found a template sequence that was between 3.7 and 7.5% different from the optimal template when using kmer searching; the V6 candidates averaged a difference of 12.5% from their optimal template. A difference of 10% between V6 fragments is comparable to a 3% difference over the full length of the gene (unpublished data); therefore a 12.5% difference is not out of line. The suffix tree searches only found the best template sequence for 25–62% of the candidate sequences. With the exception of the V6 region, the average candidate sequence found a template sequence that was between 7 and 17% different from the optimal template when using suffix tree searching; the V6 candidates averaged a difference of 37% from their optimal template. Using blastn, I found that for every region except the V14 and V19, many candidate sequences could not find a significant match to the template database (Table 2). For example, 106 (0.06%) of the V2 and 64,389 (35%) of the V6 candidates could not find a significant match. Although this may be due to the use of a large blastn word size, more sensitive searches with smaller word sizes became to slow to be practical. The blastn approach found the best template for between 17 and 63% of the candidate sequences in each region.

**Table 1:** Comparison of search methods when using the V2 and V19 candidate sequences and full-length template sequences. a

Region	Method	Speed (seqs/s)	% Correct template	% Δsimilarity (sd)^b^
V2	5-mers	118	50.6	11.7 (12.8)
	6-mers	180	68.3	7.2 (11.2)
	7-mers	280	72.6	6.1 (10.6)
	8-mers	225	72.7	6.1 (10.5)
	9-mers	202	72.4	6.1 (10.5)
	10-mers	104	71.7	6.3 (10.6)
	Suffix tree	5.5	39.4	15.3 (13.6)
	blastn	8.8	49.0	13.0 (13.8)
V19	5-mers	37	54.9	8.3 (10.0)
	6-mers	34	72.0	5.0 (8.5)
	7-mers	41	74.6	4.5 (8.2)
	8-mers	49	74.6	4.5 (8.2)
	9-mers	52	74.1	4.6 (8.3)
	10-mers	43	73.4	4.7 (8.4)
	Suffix tree	1.9	62.3	7.2 (9.9)
	blastn	0.7	63.4	6.8 (9.6)

a Data for the other regions and comparisons to region specific template sequences are provided in Tables S1 and S2.

b The average percentage difference in similarity between the correct template and the actual template returned by the search method for each candidate sequence. Smaller values indicate that more similar sequences were identified. Values in parentheses represent the standard deviation.

**Table 2:** Number of candidate sequences that did not yield a significant blast match against the full-length or region-specific template databases.

Region	Total Candidate Seqs.	Template type	Count	%
V19	186,206	Full-length	0	0.00
		Region-specific	NA	NA
V14	139,987	Full-length	0	0.00
		Region-specific	0	0.00
V12	139,987	Full-length	12	0.01
		Region-specific	12	0.01
V2	186,206	Full-length	106	0.06
		Region-specific	105	0.06
V23	186,206	Full-length	10	0.01
		Region-specific	11	0.01
V3	186,206	Full-length	432	0.23
		Region-specific	432	0.23
V4	186,206	Full-length	548	0.29
		Region-specific	546	0.29
V6	186,206	Full-length	64,389	34.6
		Region-specific	64,089	34.4
V89	77,685	Full-length	1	0.00
		Region-specific	1	0.00
V9	77,685	Full-length	14	0.02
		Region-specific	14	0.02

With respect to the time required for each approach, kmer searching outperformed the other methods regardless of the region being investigated (Table 1 and S1). In general kmer sizes of 7 or 8 were typically the fastest. Also, speed scaled with the candidate sequence length. For example, approximately 1,490 V6, 192 V23, and 52 V19 candidates sequences could be searched per second. Suffix tree searching required between 25 and 65 times longer than kmer searching. Blastn searches required between 25 and 70 times longer than kmer searching. While these speeds are implementation-dependent, the stark differences in speed and quality indicate that kmer searching was superior to other methods. While slight improvements are possible by tailoring the kmer size to the region or specific gene of interest, in general, 8-mers provided the best and fastest alignments for 16S rRNA gene fragment sequences.

I also investigated the effect of using template sequences that corresponded to the region being aligned on whether there was an improvement in accuracy or speed (Table S2). There were no significant improvements in accuracy for any of the methods when using the customized template sequences. The most noticeable effects of shorter templates were the optimal kmer size and speed. For all regions the optimal kmer size decreased from 7 to 9-mers to 5 to 7-mers. Using region-specific template sequences increased the search speed by up to 56%.

### Pairwise Alignment Methods

I investigated the accuracy and speed of the various alignment methods when the true best template sequence was selected for each candidate sequence (Tables 3 and S3). I tested the Needleman-Wunsch and Gotoh global alignment algorithms, which only differ in the number of parameters they use to penalize gaps. It was expected that Gotoh would be slower, but more accurate than the Needleman-Wunch algorithm because it uses an extra parameter. I also tested blastn, a local alignment method that approximates the Smith-Waterman algorithm [20]. The greengenes aligner currently implements blastn to carryout pairwise alignments. This approach was expected to be the fastest, but perhaps least accurate of the three methods. Furthermore, because it is a local alignment approach, it was expected to trim the ends of sequences that were sufficiently different from their template.

**Table 3:** Summary of alignment improvement for V19 candidate sequences using the blastn, Gotoh, or Needleman-Wunsch pairwise alignment algorithms when the best template was selected for each candidate sequence. a

Alignment method	Speed (seq/s)	Gap opening	Gap extension	% Δsimilarity (sd)^b^
blastn	10–12	5	2	0.42 (0.89)
		4	2	0.41 (0.87)
		3	2	0.42 (0.87)
		2	2	0.41 (0.82)
		1	2	0.43 (0.78)
		4	1	0.34 (0.68)
		3	1	0.36 (0.68)
		2	1	0.39 (0.69)
Gotoh	15–17	5	2	0.23 (0.44)
		4	2	0.24 (0.45)
		3	2	0.27 (0.48)
		2	2	0.29 (0.49)
		1	2	0.34 (0.55)
		4	1	0.25 (0.45)
		3	1	0.29 (0.49)
		2	1	0.32 (0.52)
		1	1	0.41 (0.61)
Needleman-Wunsch	21–24	5	NA	0.27 (0.49)
		4	NA	0.30 (0.51)
		3	NA	0.34 (0.55)
		2	NA	0.42 (0.62)
		1	NA	0.38 (0.60)

a Data for the other regions and comparisons to region specific template sequences are provided in Tables S3 and S4.

b The average percentage difference in similarity between the template sequence and the SILVA aligned candidate sequence and the difference in similarity between the template sequence and the candidate sequence aligned by the different implementations. Positive values indicate the candidate alignment is more similar to the template sequence and negative values are less similar. Values in parentheses indicate the standard deviation.

b blastn does not permit these gap penalties when using a match reward and mismatch penalty of 1 and the Needleman-Wunsch algorithm only takes one gap penalty parameter.

I rewarded matches and penalized mismatches with one point each and varied the gap opening and extension penalties to identify the best alignment conditions. Surprisingly, there was little variation in alignment quality regardless of the algorithm or conditions selected (Tables 3 and S3). With the exception of the V6 region, the realigned candidate sequences were, on average, 0.04–0.66 percentage points more similar to the template sequence than the SILVA-aligned candidate sequences regardless of the algorithm or penalties employed. For the V6 region, blastn produced alignments that were 1.64–1.85 percentage points worse; however the Needleman-Wunsch and Gotoh algorithms produced alignments that were 0.30 to 0.63 percentage points better. Although the effects of the different penalty schemes were minimal, the best alignments were produced using the Needleman-Wunsch algorithm with a gap penalty of 2 for all regions except the V6, which had an optimal gap penalty of 1. As expected, the blastn algorithm tended to truncate the candidate sequence alignment more frequently than the global alignment algorithms. For example, the optimal alignment conditions for the V6 region aligned each of the candidate sequences completely. In contrast, the optimal blastn condition aligned an average of 95.4% of each sequence (s.d. = 18.7%).

Universally, the Needleman-Wunsch algorithm was the fastest of the three methods followed by the Gotoh and blastn approaches. My implementation of the Needleman-Wunsch algorithm aligned between 21 and 73 sequences per second. Similar to the kmer searching implementation, these speeds were affected by candidate sequence length. Using region specific template sequences had no significant effect on alignment quality but increased the pairwise alignment speed by up to 116% (Table S4).

### Assessing the Overall Algorithm

The data in Tables 1, S1, and S2 indicated that the various search methods did not necessarily identify the best template sequence. Yet in the second set of simulations, I used the true best template to investigate the various alignment options and found that the alignments were as good as the SILVA alignments (Tables 2, S3, and S4). In the next set of simulations, I selected the best kmer size and alignment parameters for each region to evaluate the overall process (Table 4). On average the resulting alignments were between 0.61% worse and 0.34% better than the alignments generated by SILVA when using full-length template sequences. Although speed was affected by candidate sequence length, between 18 and 78 sequences were aligned per second. When using the region-specific template sequences, the alignments were between 0.07% worse and 0.40% better than the alignments generated by SILVA. The region-specific templates increased the overall alignment speed so that between 37 and 145 sequences were aligned per second.

**Table 4:** Analysis of optimal alignment settings for each variable region when using full-length, region specific, and vertical-gap filtered full-length template sequences.

Region	Template sequences	Speed (seqs/s)	% Δsimilarity (sd)^a^	% Trimmed^b^
V19	Full-length	18	0.34 (0.64)	0.17
	Region-specific	NA	NA	NA
	Vertical-gap filtered	22	0.34 (0.65)	0.17
V14	Full-length	31	0.30 (0.84)	0.20
	Region-specific	37	0.31 (0.83)	0.20
	Vertical-gap filtered	41	0.29 (0.84)	0.20
V12	Full-length	51	0.29 (1.59)	0.29
	Region-specific	79	0.40 (1.52)	0.26
	Vertical-gap filtered	88	0.32 (1.57)	0.27
V2	Full-length	58	−0.09 (1.23)	0.02
	Region-specific	100	−0.01 (1.16)	0.10
	Vertical-gap filtered	105	−0.09 (1.23)	0.02
V23	Full-length	43	0.07 (0.91)	0.02
	Region-specific	64	0.14 (0.86)	0.23
	Vertical-gap filtered	65	0.07 (0.91)	0.02
V3	Full-length	69	−0.18 (1.50)	0.00
	Region-specific	122	−0.06 (1.35)	0.27
	Vertical-gap filtered	151	−0.16 (1.49)	0.00
V4	Full-length	61	−0.19 (1.00)	0.00
	Region-specific	100	−0.07 (0.75)	0.00
	Vertical-gap filtered	109	−0.19 (1.00)	0.00
V6	Full-length	78	−0.61 (3.63)	0.02
	Region-specific	145	−0.02 (2.92)	0.44
	Vertical-gap filtered	204	−0.64 (3.66)	0.02
V89	Full-length	45	0.09 (0.78)	0.13
	Region-specific	70	0.12 (0.75)	0.12
	Vertical-gap filtered	64	0.09 (0.78)	0.13
V9	Full-length	61	0.01 (1.24)	0.21
	Region-specific	100	0.08 (1.14)	0.17
	Vertical-gap filtered	102	0.01 (1.24)	0.21

a See description for Table 3.

b The percentage of sequences where less than 95% of the bases were aligned to the template sequence.

### Effect of Full-Length Alignment Length

This study used a 50,000-column MSA alignment for a gene that is approximately 1,500-bp long. In comparison, the greengenes alignment has 7,682 columns. The extended SILVA alignment has been justified by a desire to include archaeal 16S rRNA and eukaryotic 18S rRNA gene sequences as well as to provide sufficient padding for new sequences that may have long insertions. To simulate a greengenes-like alignment, I removed any column from the SILVA alignment only contained gap characters. This produced a 6,806-column alignment. Using the transformed template alignment sequences, I found that the alignment quality did not vary considerably from the results obtained with the 50,000-column MSA (Table 4). Furthermore, although the same number of bases were represented in each sequence, the speeds observed using vertical gap-filtered MSA were up to 160% faster than using the full 50,000 character alignment.

### Comparison to the Greengenes Implementations

I implemented the approaches that have been used by greengenes to assess the sensitivity of the overall algorithm. Using the original approach of employing 7-mer searching combined with blast alignments I found that with the exception of the V6 region, the alignments were between 0.19% worse and 0.36% better than the SILVA alignments (Table 5). These values are somewhat deceiving as I found that, with the exception of the V6 region, between 0.8 and 4.8% of the sequence in each region had less than 95% of its bases aligned. This version of the algorithm aligned between 15 and 25 sequences per second. Although this is considerably slower than what I observed using with the optimal conditions it is considerably faster than the 10 sequences per minute (i.e. 0.17 sequences per second) that was described previously [19]. To mimic the current greengenes implementation, I used blastn to find the closest match. This slightly improved the overall alignments over the original greengenes implementation so that, with the exception of the V6 region, the average alignment was between 0.27% worse and 0.69% better than the SILVA alignments. Again, these values are deceiving, as between 1.1 and 5.4% of the sequences had fewer than 95% of their bases aligned in each sequence. The greengenes aligner approach suffered when aligning the V6 region because of is dependence on blastn. When using 7-mers and blastn the candidate sequences averaged 4.6% (s.d. = 19.5%) worse than the SILVA candidate sequences and more than 18.0% of the sequences had less than 95% of its bases aligned. Considering 35% of the V6 candidate sequences could not be matched to a template via blastn, the current greengenes implementation was ineffective.

**Table 5:** Analysis of two versions of the greengenes aligner when aligning various regions to full-length SILVA-aligned template sequences.

Region	greengenes version	Speed (seqs/s)	% Δsimilarity (sd)^a^	% Trimmed^b^
V19	Original^c^	15	0.31 (0.83)	3.09
	Current^d^	0.6	0.37 (0.82)	3.06
V14	Original	18	0.26 (1.19)	4.82
	Current	1.5	0.39 (1.14)	4.27
V12	Original	18	0.36 (4.51)	4.51
	Current	4.5	0.69 (2.73)	5.40
V2	Original	19	−0.09 (0.91)	0.91
	Current	5.0	0.27 (2.77)	2.04
V23	Original	17	0.07 (1.15)	2.16
	Current	2.2	0.25 (1.27)	2.08
V3	Original	20	−0.02 (1.85)	2.18
	Current	7.3	0.00 (4.41)	2.52
V4	Original	19	−0.19 (1.03)	0.77
	Current	5.9	−0.27 (4.44)	1.10
V6	Original	26	−4.58 (19.5)	18.0
	Current	12	−28.4 (39.9)	37.7
V89	Original	20	0.14 (1.07)	1.99
	Current	2.0	0.27 (1.08)	1.96
V9	Original	21	0.16 (1.76)	2.09
	Current	3.0	0.29 (1.99)	1.82

a, b See descriptions for Table 4.

c Searching with 7-mers and using blastn to align.

d Searching and aligning with blastn.

## Discussion

A critical step in analyzing DNA sequences generated from community surveys is generating a MSA. Here I described and validated a variation of the greengenes and SILVA aligners and showed that this aligner quickly generates a high-quality alignment. Also, although investigators are encouraged to perform similar types of experiments to optimize the alignment conditions for their region of interest, the kmer search and Needlema-Wunsch alignment approach was robust to perturbations in their settings. Interestingly, whereas other aligners appear to use multiple template sequences to align one candidate sequence, this alignment algorithm only requires one reference sequence per candidate sequence and does require explicit knowledge of the 16S rRNA secondary structure.

An important consideration in selecting a reference alignment is its underlying quality, yet manually curating a reference alignment is a tedious and painstaking process. It is a common practice to mask hypervariable regions when generating a deep-level phylogeny [21]. Such a practice enables one to ignore the alignment of the masked out regions, which is typically where the problematic areas are located. However, when assigning sequences to OTUs or using phylogenies for community-based hypothesis tests, the fine level of detail contained within these variable regions is significant and should not be removed. The original Lane mask removes, on average, 14% of the bases from a full-length sequence alignment of *E. coli*'s full-length 16S rRNA gene [21]. Considering the typical pyrosequencing-based study focuses on these variable regions and the sequence reads generated by pyrosequencing tend to be shorter than 250 bp, it is important that these regions not be discarded. While some have mistakenly used such masks prior to performing other analyses [4], [6], [22]–[25], a better practice would be to manually curate the reference alignment in these regions and to include all of the data. Ultimately, the quality of an alignment will only be as good as reference alignment, regardless of the algorithm.

Because this aligner is not tied to a particular database alignment, this aligner can be used with any reference aligner whether the DNA represent rRNA genes or protein coding genes [e.g. 26]. The first step in such an analysis is to generate a *de novo* multiple sequence alignment using software such as Clustal, MUSCLE, or MAFFT. Second, the new reference alignment should be curated to insure positional homology across the alignment. Third, unaligned sequences could then be aligned to the reference alignment using a variety of parameters. Finally, manual inspection of the newly aligned sequences should reveal the optimal parameters. Considering the general lack of sensitivity of the method to variations in the various parameters it is likely that the parameters described here will work for other genes.

As microbial ecologists continue to generate massive data sets, it is important to continually refine and validate every step in the analysis pipeline. Central to this ideal is the availability of open source software and peer-reviewed methods. The combined generation of a fast, parallelized, open source, and flexible aligner with the simulations performed in this study demonstrate that this tool will be a valuable contribution to future investigations.

## Methods

### Selection of Sequences Used in Analysis

I obtained the SSURef (Release 96) alignment from the SILVA database on October 14, 2008, which contained 271,543 bacterial 16S rRNA gene sequences longer than 1,200 bp. Aligned sequences were downloaded from the greengene and RDP databases on November 16, 2008. The SILVA database was selected because it was the longest and visual inspection of the alignment suggested that it had the highest overall quality (Fig. 2). Using ARB, I removed any sequence that affiliated with mitochondria or chloroplasts or were flagged as being of poor alignments, having more than five ambiguous bases, or appearing chimeric. This screen resulted in a collection of 243,472 high-quality aligned sequences. I dereplicated these sequences to obtain 222,086 unique sequences. Finally, I cross-matched accessions between the SILVA, greengenes, and RDP databases to obtain a collection of 200,433 sequences.

Because the SILVA reference alignment (i.e. the SEED) is not publicly available, I attempted to replicate the SEED database. First, I parsed the 200,433 sequences to identify those sequences that had an alignment quality score (i.e. ARB database field ‘align_quality_slv’) of 100. Next, I identified those sequences in this pool that started by *E. coli* position 28 and ended after position 1491. Sequences beginning before or ending after these coordinates were trimmed. The resulting collection of 14,227 sequences represented my full-length template database. The other 186,206 sequences represented the candidate sequence collection.

### Generation of Region Specific Datasets

I selected 10 regions within the 16S rRNA gene for my simulations. While maintaining the overall 50,000-character alignment, I excised regions V19 (*E. coli* positions 28–1491), V12 (28–337), V14 (28–784), V2 (100–337), V23 (100–514), V3 (357–514), V4 (578–784), V6 (986–1045), V89 (1100–1491), and V9 (1300–1491). There were 186,206 candidate sequences for analyzing the V19, V2, V23, V3, V4, and V6 datasets. Because not all sequences extended through the first and ninth variable regions, I further screened these sequences to generate a collection of 139,987 candidate sequences for analyzing the V12 and V14 datasets and a collection of 77,685 sequences for the V89 and V9 datasets. These regions were selected because they are tractable by Sanger (V19, V14), 454 GS-FLX (V2, V3, V4, V6, V9), 454 Titanium (V12, V23, V89), and Illumina (V3, V6) sequencing technologies. Many of these regions have also been used in published studies: V19 [e.g. 2], V14 [e.g. 1], V2 [e.g. 4], V3 [e.g. 27], V4 [15], V6 [e.g. 3], and V9 [e.g. 28]. Considering there are myriad permutations of these regions, these provided a generous coverage of the 16S rRNA gene.

### Permutations of the Greengenes Search Step

I tested three search options: blastn, kmer searching, and suffix tree searching (Fig. 1). First, I used blastn as made available from NCBI with a word size of 28, match reward of 1, mismatch penalty of -1. These settings are comparable to those used in megablast and were needed to make the search times competitive with the other methods. Other parameters used for the blast included returning one result in the tabular format (–b 1 –m 8). Second, I used kmer searching with word sizes ranging between 5 and 10; smaller and larger words did not improve the searches and were considerably slower. The kmer-searching algorithm involved generating a lookup table where the keys in the table corresponded to all possible 4^K^ kmers. These keys pointed to a list of template sequence identifiers. Next, the software identified all possible kmers for a candidate sequence and counts the number of kmers each template sequence shares with the candidate sequence. An analogous procedure is used in the initial steps of the MUSCLE algorithm [9]. The template with the most kmers in common was then used for further analysis. Third, I generated a suffix tree for each template sequence [29]. Using the string-to-string block-move algorithm I identified the template suffix tree that broke the candidate sequence into the fewest suffix sequences [30].

### Permutations of the Greengenes Pairwise Alignment Step

I tested three pairwise alignment methods: blastn [20] and the Needleman-Wunsch [31] and Gotoh [32], [33] global alignment algorithms (Fig. 1). While my implementation of the aligner permits changing the match and mismatch scores, I chose to use +1 and -1 in all simulations. I used combinations of gap opening penalties of 5, 4, 3, 2, and 1 with gap extension penalties of 2 and 1. These values were selected to overlap as much as possible with the combinations that are implemented in the nucleotide-based BLAST program. The bl2seq BLAST program was used to obtain pairwise alignments using BLAST with the default word size of 11 (-W 11). To improve the Needleman-Wunsch and Gotoh alignments at the ends of sequences that do not fully overlap, I followed the end-space free variant algorithm described by Gusfield [34].

### Benchmarking of Methods

To assess the ability of each method to properly identify the correct reference sequence, I calculated the raw similarity between each candidate sequence and all template sequences. In these similarity calculations, the comparison of a gap with a base is counted as a mismatch and the comparison between a pair of gaps does not factor into the calculation. These similarity scores were calculated for each region under consideration because the template for a candidate fragment would not necessarily be the same as the template for the full-length candidate sequence. The template sequence that was most similar to the candidate sequence was considered the true best template sequence.

All simulations were run on a MacPro computer with 9 GB 667 MHz DDR2 RAM and 2×3 GHz Dual-Core Intel Xenon processors. To insure that each analysis was running at optimal speed, I only used 3 processors at a time and each analysis only used one processor. At no time did RAM utilization approach 9 GB. All source code was written in C++ and compiled using the –O3 compiler optimization flag.

### Availability of Software

The aligner described here is freely available and provided within the mothur software package as source code or as a Windows executable (http://www.mothur.org) [35]. The defaults within mothur include using kmer searching with a word size of 8 and using the Needleman-Wunsch algorithm for pairwise alignments with a gap penalty of −2; however, all of the methods can be selected by users with the ability to modify any of the match and mismatch scores and gap penalties. Although not used in this study, the mothur implementation enables users to use multiple processors to accelerate the alignment. The mothur implementation requires that the user input FASTA-formatted files containing their candidate sequences and template database. Example template databases, including the one used in this study, are available from the mothur website.

## Supporting Information

Table S1 Comparison of search methods when using various regions extracted from candidate sequences and full-length template sequences.
(0.07 MB PDF)

Table S2 Comparison of search methods when using various regions extracted from candidate sequences and region-specific template sequences.
(0.06 MB PDF)

Table S3 Summary of alignment improvement for various regions extracted from candidate sequences using the blastn, Gotoh, or Needleman-Wunsch pairwise alignment algorithms when the best full-length template was selected for each candidate sequence.
(0.11 MB PDF)

Table S4 Summary of alignment improvement for various regions extracted from candidate sequences using the blastn, Gotoh, or Needleman-Wunsch pairwise alignment algorithms when the best region-specific template was selected for each candidate sequence.
(0.10 MB PDF)

## References

[1] Schloss PD, Handelsman J (2006). Toward a census of bacteria in soil.. PLoS Comp Biol.

[2] Eckburg PB, Bik EM, Bernstein CN, Purdom E, Dethlefsen L (2005). Diversity of the human intestinal microbial flora.. Science.

[3] Sogin ML, Morrison HG, Huber JA, Welch DM, Huse SM (2006). Microbial diversity in the deep sea and the underexplored “rare biosphere”.. Proc Natl Acad Sci U S A.

[4] Turnbaugh PJ, Hamady M, Yatsunenko T, Cantarel BL, Duncan A (2009). A core gut microbiome in obese and lean twins.. Nature.

[5] Schloss PD, Handelsman J (2005). Introducing DOTUR, a computer program for defining operational taxonomic units and estimating species richness.. Appl Environ Microbiol.

[6] Fierer N, Hamady M, Lauber CL, Knight R (2008). The influence of sex, handedness, and washing on the diversity of hand surface bacteria.. Proc Natl Acad Sci U S A.

[7] Chenna R, Sugawara H, Koike T, Lopez R, Gibson TJ (2003). Multiple sequence alignment with the Clustal series of programs.. Nucleic Acids Res.

[8] Katoh K, Asimenos G, Toh H (2009). Multiple alignment of DNA sequences with MAFFT.. Methods Mol Biol.

[9] Edgar RC (2004). MUSCLE: a multiple sequence alignment method with reduced time and space complexity.. BMC Bioinformatics.

[10] Huber JA, Mark Welch DB, Morrison HG, Huse SM, Neal PR (2007). Microbial population structures in the deep marine biosphere.. Science.

[11] Antonopoulos DA, Huse SM, Morrison HG, Schmidt TM, Sogin ML (2009). Reproducible community dynamics of the gastrointestinal microbiota following antibiotic perturbation.. Infect Immun.

[12] Sun Y, Cai Y, Liu L, Yu F, Farrell ML (2009). ESPRIT: estimating species richness using large collections of 16S rRNA pyrosequences.. Nucleic Acids Res.

[13] Gardner PP, Wilm A, Washietl S (2005). A benchmark of multiple sequence alignment programs upon structural RNAs.. Nucleic Acids Res.

[14] Nawrocki EP, Kolbe DL, Eddy SR (2009). Infernal 1.0: inference of RNA alignments.. Bioinformatics.

[15] Cole JR, Wang Q, Cardenas E, Fish J, Chai B (2009). The Ribosomal Database Project: improved alignments and new tools for rRNA analysis.. Nucleic Acids Res.

[16] Ludwig W, Strunk O, Westram R, Richter L, Meier H (2004). ARB: A software environment for sequence data.. Nucleic Acids Res.

[17] Pruesse E, Quast C, Knittel K, Fuchs BM, Ludwig W (2007). SILVA: a comprehensive online resource for quality checked and aligned ribosomal RNA sequence data compatible with ARB.. Nucleic Acids Res.

[18] DeSantis TZ, Hugenholtz P, Larsen N, Rojas M, Brodie EL (2006). Greengenes, a chimera-checked 16S rRNA gene database and workbench compatible with ARB.. Appl Environ Microbiol.

[19] DeSantis TZ, Hugenholtz P, Keller K, Brodie EL, Larsen N (2006). NAST: a multiple sequence alignment server for comparative analysis of 16S rRNA genes.. Nucleic Acids Res.

[20] Altschul SF, Madden TL, Schaffer AA, Zhang J, Zhang Z (1997). Gapped BLAST and PSI-BLAST: a new generation of protein database search programs.. Nucleic Acids Res.

[21] Lane DJ, Stackebrandt E, Goodfellow M, (1991). 16S/23S rRNA sequencing.. Nucleic Acid Techniques in Bacterial Systematics.

[22] Jones RT, Robeson MS, Lauber CL, Hamady M, Knight R (2009). A comprehensive survey of soil acidobacterial diversity using pyrosequencing and clone library analyses.. ISME J.

[23] Lauber CL, Hamady M, Knight R, Fierer N (2009). Pyrosequencing-based assessment of soil pH as a predictor of soil bacterial community structure at the continental scale.. Appl Environ Microbiol.

[24] Bowers RM, Lauber CL, Wiedinmyer C, Hamady M, Hallar AG (2009). Characterization of airborne microbial communities at a high elevation site and their potential to act as atmospheric ice nuclei.. Appl Environ Microbiol.

[25] Crawford PA, Crowley JR, Sambandam N, Muegge BD, Costello EK (2009). Regulation of myocardial ketone body metabolism by the gut microbiota during nutrient deprivation.. Proc Natl Acad Sci U S A.

[26] Schellenberg J, Links MG, Hill JE, Dumonceaux TJ, Peters GA (2009). Pyrosequencing of the chaperonin-60 universal target as a tool for determining microbial community composition.. Appl Environ Microbiol.

[27] Dethlefsen L, Huse S, Sogin ML, Relman DA (2008). The pervasive effects of an antibiotic on the human gut microbiota, as revealed by deep 16S rRNA sequencing.. PLoS Biol.

[28] Roesch LFW, Fulthorpe RR, Riva A, Casella G, Hadwin AKM (2007). Pyrosequencing enumerates and contrasts soil microbial diversity.. ISME J.

[29] Ukkonen E (1995). Online construction of suffix trees.. Algorithmica.

[30] Tichy WF (1984). The string-to-string correction problem with block moves.. ACM Trans Comput Syst.

[31] Needleman SB, Wunsch CD (1970). A general method applicable to the search for similarities in the amino acid sequence of two proteins.. J Mol Biol.

[32] Myers EW, Miller W (1988). Optimal alignments in linear space.. Comput Appl Biosci.

[33] Gotoh O (1982). An improved algorithm for matching biological sequences.. J Mol Biol.

[34] Gusfield D (1997). Algorithms on strings, trees, and sequences: computer science and computational biology..

[35] Schloss PD, Westcott SL, Ryabin T, Hall JR, Hartmann M (2009). Introducing mothur: Open source, platform-independent, community-supported software for describing and comparing microbial communities.. Appl Environ Microbiol.

