# Supplementary material for: A High-Throughput DNA Sequence Aligner for Microbial Ecology Studies
Source: PLoS One. 2009 Dec 14;4(12):e8230. doi: 10.1371/journal.pone.0008230 (PMC2788221; doi:10.1371/journal.pone.0008230)
Supplement: Table S1 — Comparison of search methods when using various regions extracted from candidate sequences and full-length template sequences. (0.07 MB PDF) [file pone.0008230.s001.pdf]

Table S1. Comparison of search methods when using various regions extracted from candidate sequences and full-length template sequences.

Region	Method	Speed (seqs/s)	% Correct template	% Δsimilarity (sd)[a]
V19	5-mers	37	54.9	8.3 (10.0)
	6-mers	34	72.0	5.0 (8.5)
	7-mers	41	74.6	4.5 (8.2)
	8-mers	49	74.6	4.5 (8.2)
	9-mers	52	74.1	4.6 (8.3)
	10-mers	43	73.4	4.7 (8.4)
	Suffix tree	1.9	62.3	7.2 (9.9)
	blastn	0.7	63.4	6.8 (9.6)
V14	5-mers	54	55.3	8.9 (10.7)
	6-mers	65	71.9	5.3 (9.1)
	7-mers	90	73.5	5.0 (8.9)
	8-mers	107	73.6	5.0 (8.9)
	9-mers	90	73.3	5.0 (8.9)
	10-mers	81	72.9	5.1 (8.9)
	Suffix tree	3	55.2	9.5 (11.4)
	blastn	2	60.6	8.3 (11.1)
V12	5-mers	97	47.7	12.7 (13.2)
	6-mers	146	64.2	8.3 (12.0)
	7-mers	211	67.3	7.6 (11.7)
	8-mers	243	67.6	7.5 (11.6)
	9-mers	210	67.6	7.5 (11.6)
	10-mers	103	67.4	7.5 (11.6)
	Suffix tree	4	41.1	15.4 (14.2)
	blastn	7	48.0	13.5 (14.2)
V2	5-mers	118	50.6	11.7 (12.8)
	6-mers	180	68.3	7.2 (11.2)
	7-mers	280	72.6	6.1 (10.6)
	8-mers	225	72.7	6.1 (10.5)
	9-mers	202	72.4	6.1 (10.5)
	10-mers	104	71.7	6.3 (10.6)
	Suffix tree	5.5	39.4	15.3 (13.6)
	blastn	8.8	49.0	13.0 (13.8)
V23	5-mers	77	52.4	10.2 (11.6)
	6-mers	109	70.7	6.1 (10.1)
	7-mers	159	73.1	5.5 (9.7)
	8-mers	192	73.4	5.4 (9.6)
	9-mers	178	73.8	5.3 (9.5)
	10-mers	95	73.3	5.4 (9.6)
	Suffix tree	4	47.2	12.1 (12.5)
	blastn	3	55.7	10.3 (12.5)
V3	5-mers	174	46.8	11.6 (11.9)
	6-mers	277	70.0	6.3 (10.3)
	7-mers	428	74.0	5.5 (9.8)
	8-mers	455	75.8	5.1 (9.6)
	9-mers	323	76.5	4.9 (9.5)
	10-mers	122	76.7	4.9 (9.5)
	Suffix tree	8	26.6	17.0 (12.0)
	blastn	14	32.8	15.7 (12.9)
V4	5-mers	133	50.9	9.2 (10.1)
	6-mers	201	72.4	4.8 (8.2)
	7-mers	315	75.1	4.2 (7.7)
	8-mers	388	77.2	3.8 (7.4)
	9-mers	309	77.4	3.8 (7.4)
	10-mers	130	77.4	3.7 (7.3)
	Suffix tree	5.8	33.3	13.9 (10.8)
	blastn	1.1	40.7	12.7 (11.9)
V6	5-mers	449	42.8	26.8 (24.6)
	6-mers	792	63.8	15.8 (21.8)
	7-mers	1400	68.4	13.6 (20.8)
	8-mers	1490	70.3	12.7 (20.3)
	9-mers	644	70.6	12.5 (20.2)

	10-mers	170	70.4	12.6 (20.2)
	Suffix tree	12	25.1	36.9 (23.4)
	blastn	31	17.1	54.5 (29.1)
V89	5-mers	79	51.6	9.1 (10.2)
	6-mers	104	70.7	5.2 (8.7)
	7-mers	142	75.0	4.3 (8.1)
	8-mers	168	76.4	4.1 (7.9)
	9-mers	163	76.6	4.0 (7.8)
	10-mers	102	76.4	4.0 (7.8)
	Suffix tree	5.4	40.8	11.7 (10.6)
	blastn	2.4	47.8	10.5 (10.8)
V9	5-mers	135	47.0	9.9 (10.2)
	6-mers	195	70.3	5.3 (8.7)
	7-mers	263	74.8	4.4 (8.1)
	8-mers	278	76.6	4.1 (7.8)
	9-mers	239	77.0	3.9 (7.7)
	10-mers	117	77.0	3.9 (7.7)
	Suffix tree	8.8	28.1	14.1 (10.1)
	blastn	3.4	33.1	13.3 (10.5)

a The average percentage difference in similarity between the correct template and the actual template returned by the search method for each candidate sequence. Smaller values indicate that more similar sequences were identified. Values in parentheses represent the standard deviation.
