# Supplementary material for: A High-Throughput DNA Sequence Aligner for Microbial Ecology Studies
Source: PLoS One. 2009 Dec 14;4(12):e8230. doi: 10.1371/journal.pone.0008230 (PMC2788221; doi:10.1371/journal.pone.0008230)
Supplement: Table S2 — Comparison of search methods when using various regions extracted from candidate sequences and region-specific template sequences. (0.06 MB PDF) [file pone.0008230.s002.pdf]

Table S2. Comparison of search methods when using various regions extracted from candidate sequences and region-specific template sequences.

Region	Method	Speed (seqs/s)	% Correct template	% Δsimilarity (sd)[a]
V14	5-mers	70	66.6	6.5 (9.8)
	6-mers	81	73.7	5.0 (8.9)
	7-mers	104	74.2	4.8 (8.8)
	8-mers	121	74.0	4.9 (8.8)
	9-mers	122	73.5	5.0 (8.9)
	10-mers	85	73.0	5.1 (8.9)
	Suffix tree	4	55.7	9.4 (11.4)
	blastn	2	60.6	8.3 (11.1)
V12	5-mers	136	66.5	7.7 (11.6)
	6-mers	178	69.6	7.0(11.3)
	7-mers	216	69.1	7.1 (11.4)
	8-mers	211	68.5	7.2 (11.4)
	9-mers	244	67.9	7.4 (11.5)
	10-mers	128	67.6	7.4 (11.5)
	Suffix tree	7	42.9	15.0 (14.3)
	blastn	8	48.0	13.5 (14.2)
V2	5-mers	196	68.1	7.3 (11.4)
	6-mers	281	73.7	5.9 (10.4)
	7-mers	372	74.3	5.7 (10.3)
	8-mers	430	73.6	5.8 (10.4)
	9-mers	353	72.5	6.1 (10.5)
	10-mers	144	71.8	6.3 (10.6)
	Suffix tree	8	40.7	15.0 (13.6)
	blastn	9	49.0	13.0 (13.8)
V23	5-mers	127	72.4	5.7 (9.8)
	6-mers	161	75.9	4.9 (9.2)
	7-mers	201	75.2	5.0 (9.3)
	8-mers	226	74.6	5.1 (9.4)
	9-mers	206	74.0	5.3 (9.4)
	10-mers	113	73.4	5.4 (9.5)
	Suffix tree	6	48.3	11.9 (12.5)
	blastn	3	55.7	10.3 (12.5)
V3	5-mers	321	78.8	4.5 (9.1)
	6-mers	434	78.8	4.4 (9.1)
	7-mers	512	78.5	4.5 (9.2)
	8-mers	523	78.0	4.6 (9.3)
	9-mers	366	77.5	4.7 (9.3)
	10-mers	138	77.0	4.8 (9.4)
	Suffix tree	17	28.6	16.6 (12.2)
	blastn	18	32.8	15.7 (12.9)
V4	5-mers	268	79.4	3.5 (7.2)
	6-mers	351	81.1	3.2 (6.9)
	7-mers	437	80.0	3.3 (7.0)
	8-mers	501	79.2	3.4 (7.0)
	9-mers	399	78.6	3.5 (7.1)
	10-mers	153	77.6	3.7 (7.3)
	Suffix tree	13	34.9	13.6 (10.9)
	blastn	13	40.7	12.7 (11.9)
V6	5-mers	1633	73.9	10.9 (19.1)
	6-mers	2140	73.7	11.1 (19.3)
	7-mers	2328	73.0	11.4 (19.5)
	8-mers	1648	72.2	11.8 (19.7)
	9-mers	584	71.6	12.1 (19.9)
	10-mers	147	70.9	12.4 (20.1)
	Suffix tree	28	27.0	36.1 (23.9)
	blastn	50	17.1	54.4 (29.1)
V89	5-mers	117	74.0	4.6 (8.3)
	6-mers	136	77.6	3.9 (7.8)
	7-mers	160	78.2	3.7 (7.6)
	8-mers	175	77.9	3.8 (7.6)
	9-mers	161	77.4	3.8 (7.7)

	10-mers	96	76.8	3.9 (7.7)
	Suffix tree	8	41.6	11.5 (10.6)
	blastn	3	47.8	10.5 (10.8)
V9	5-mers	235	78.9	3.7 (7.6)
	6-mers	274	79.8	3.5 (7.4)
	7-mers	306	79.3	3.5 (7.4)
	8-mers	311	78.9	3.6 (7.4)
	9-mers	231	78.1	3.7 (7.6)
	10-mers	100	77.5	3.8 (7.6)
	Suffix tree	14	29.5	13.9 (10.2)
	blastn	4	33.1	13.3 (10.5)

a The average percentage difference in similarity between the correct template and the actual template returned by the search method for each candidate sequence. Smaller values indicate that more similar sequences were identified. Values in parentheses represent the standard deviation.
