# Supplementary material for: A High-Throughput DNA Sequence Aligner for Microbial Ecology Studies
Source: PLoS One. 2009 Dec 14;4(12):e8230. doi: 10.1371/journal.pone.0008230 (PMC2788221; doi:10.1371/journal.pone.0008230)
Supplement: Table S3 — Summary of alignment improvement for various regions extracted from candidate sequences using the blastn, Gotoh, or Needleman-Wunsch pairwise alignment algorithms when the best full-length template was selected for each candidate sequence. (0.11 MB PDF) [file pone.0008230.s003.pdf]

Table S3. Summary of alignment improvement for various regions extracted from candidate sequences using the blastn, Gotoh, or Needleman-Wunsch pairwise alignment algorithms when the best full-length template was selected for each candidate sequence.

Region	Alignment method	Speed (seq/s)	Gap opening	Gap extension	% Δsimilarity (sd)[a]
V19	blastn	10-12	5	2	0.42 (0.9)
			4	2	0.41 (0.9)
			3	2	0.42 (0.9)
			2	2	0.41 (0.8)
			1	2	0.43 (0.8)
			4	1	0.34 (0.7)
			3	1	0.36 (0.7)
			2	1	0.39 (0.7)
	Gotoh	15-17	5	2	0.23 (0.4)
			4	2	0.24 (0.5)
			3	2	0.27 (0.5)
			2	2	0.29 (0.5)
			1	2	0.34 (6.0)
			4	1	0.25 (0.5)
			3	1	0.29 (0.5)
			2	1	0.32 (0.5)
			1	1	0.41 (0.6)
	Needleman-Wunsch	21-24	5	NA	0.27 (0.5)
			4	NA	0.30 (0.5)
			3	NA	0.34 (0.6)
			2	NA	0.42 (0.6)
			1	NA	0.38 (0.6)
V14	blastn	10-11	5	2	0.42 (1.2)
			4	2	0.41 (1.1)
			3	2	0.42 (1.1)
			2	2	0.40 (1.0)
			1	2	0.42 (1.0)
			4	1	0.31 (0.9)
			3	1	0.34 (0.8)
			2	1	0.37 (0.8)
	Gotoh	12-13	5	2	0.22 (0.5)
			4	2	0.23 (0.5)
			3	2	0.26 (0.6)
			2	2	0.28 (0.6)
			1	2	0.35 (0.7)
			4	1	0.24 (0.5)
			3	1	0.28 (0.6)
			2	1	0.32 (0.6)
			1	1	0.43 (0.7)
	Needleman-Wunsch	16-18	5	NA	0.16 (0.6)
			4	NA	0.30 (0.6)
			3	NA	0.35 (0.7)
			2	NA	0.44 (0.7)
			1	NA	0.41 (0.7)
V12	blastn	11-13	5	2	0.63 (1.9)
			4	2	0.62 (1.9)
			3	2	0.64 (1.8)
			2	2	0.63 (1.7)
			1	2	0.66 (1.6)
			4	1	0.50 (1.5)
			3	1	0.57 (1.5)
			2	1	0.61 (1.5)
	Gotoh	36-37	5	2	0.36 (1.0)
			4	2	0.37 (1.0)
			3	2	0.43 (1.0)
			2	2	0.46 (1.1)
			1	2	0.56 (1.2)
			4	1	0.38 (1.0)
			3	1	0.46 (1.1)
			2	1	0.51 (1.1)

			1	1	0.65 (1.2)
	Needleman-Wunsch	40-46	5	NA	0.43 (1.1)
			4	NA	0.49 (1.1)
			3	NA	0.55 (1.2)
			2	NA	0.66 (1.2)
		10	1	NA	0.64 (1.2)
V2	blastn		5	2	0.29 (1.3)
			4	2	0.29 (1.2)
			3	2	0.29 (1.1)
			2	2	0.29 (1.0)
			1	2	0.32 (1.0)
			4	1	0.25 (0.9)
			3	1	0.28 (1.0)
			2	1	0.30 (1.0)
	Gotoh	46-47	5	2	0.13 (0.6)
			4	2	0.14 (0.6)
			3	2	0.16 (0.6)
			2	2	0.18 (0.7)
			1	2	0.22 (0.7)
			4	1	0.15 (0.6)
			3	1	0.18 (0.7)
			2	1	0.21 (0.7)
			1	1	0.29 (0.8)
	Needleman-Wunsch	55-59	5	NA	0.16 (0.7)
			4	NA	0.18 (0.7)
			3	NA	0.21 (0.7)
			2	NA	0.29 (0.8)
			1	NA	0.29 (0.8)
V23	blastn	10-11	5	2	0.29 (1.0)
			4	2	0.28 (1.0)
			3	2	0.28 (0.9)
			2	2	0.28 (0.9)
			1	2	0.31 (0.8)
			4	1	0.23 (0.7)
			3	1	0.25 (0.7)
			2	1	0.28 (0.7)
	Gotoh	36-37	5	2	0.15 (0.5)
			4	2	0.15 (0.5)
			3	2	0.17 (0.5)
			2	2	0.19 (0.5)
			1	2	0.23 (0.6)
			4	1	0.16 (0.5)
			3	1	0.19 (0.5)
			2	1	0.22 (0.5)
			1	1	0.30 (0.6)
	Needleman-Wunsch	44-49	5	NA	0.16 (0.6)
			4	NA	0.18 (0.6)
			3	NA	0.22 (0.6)
			2	NA	0.30 (0.6)
			1	NA	0.28 (0.7)
V3	blastn	9-11	5	2	0.21 (1.3)
			4	2	0.21 (1.3)
			3	2	0.22 (1.3)
			2	2	0.21 (1.2)
			1	2	0.24 (1.2)
			4	1	0.16 (1.0)
			3	1	0.17 (0.9)
			2	1	0.19 (0.9)
	Gotoh	61-62	5	2	0.11 (0.5)
			4	2	0.11 (0.5)
			3	2	0.12 (0.5)
			2	2	0.13 (0.6)
			1	2	0.17 (0.6)
			4	1	0.12 (0.5)
			3	1	0.13 (0.6)
			2	1	0.16 (0.6)

			1	1	0.21 (0.6)
	Needleman-Wunsch	69-73	5	NA	0.09 (0.8)
			4	NA	0.11 (0.7)
			3	NA	0.15 (0.7)
			2	NA	0.20 (0.7)
			1	NA	0.17 (0.7)
V4	blastn	11-13	5	2	0.10 (0.5)
			4	2	0.10 (0.5)
			3	2	0.10 (0.5)
			2	2	0.10 (0.5)
			1	2	0.11 (0.5)
			4	1	0.09 (0.5)
			3	1	0.10 (0.5)
			2	1	0.11 (0.5)
	Gotoh	46-47	5	2	0.04 (0.3)
			4	2	0.04 (0.3)
			3	2	0.04 (0.3)
			2	2	0.05 (0.3)
			1	2	0.06 (0.3)
			4	1	0.04 (0.3)
			3	1	0.05 (0.3)
			2	1	0.06 (0.3)
			1	1	0.12 (0.4)
	Needleman-Wunsch	55-56	5	NA	0.05 (0.3)
			4	NA	0.05 (0.3)
			3	NA	0.06 (0.3)
			2	NA	0.12 (0.4)
			1	NA	0.10 (0.4)
V6	blastn	10-12	5	2	-1.85 (14)
			4	2	-1.81 (14)
			3	2	-1.72 (14)
			2	2	-1.64 (14)
			1	2	-1.75 (14)
			4	1	-1.75 (14)
			3	1	-1.65 (14)
			2	1	-1.75 (14)
	Gotoh	63-64	5	2	0.30 (1.9)
			4	2	0.33 (1.8)
			3	2	0.40 (1.8)
			2	2	0.43 (1.8)
			1	2	0.48 (1.8)
			4	1	0.36 (1.7)
			3	1	0.43 (1.7)
			2	1	0.47 (1.7)
			1	1	0.60 (1.9)
	Needleman-Wunsch	67-70	5	NA	0.39 (1.8)
			4	NA	0.43 (1.8)
			3	NA	0.48 (1.8)
			2	NA	0.59 (1.9)
			1	NA	0.63 (1.9)
V89	blastn	10-11	5	2	0.24 (1.0)
			4	2	0.24 (0.9)
			3	2	0.25 (0.9)
			2	2	0.25 (0.9)
			1	2	0.25 (0.9)
			4	1	0.22 (0.9)
			3	1	0.23 (0.9)
			2	1	0.23 (0.8)
	Gotoh	35-36	5	2	0.15 (0.6)
			4	2	0.15 (0.6)
			3	2	0.17 (0.6)
			2	2	0.17 (0.6)
			1	2	0.19 (0.6)
			4	1	0.15 (0.6)
			3	1	0.17 (0.6)
			2	1	0.18 (0.6)

			1	1	0.21 (0.6)
	Needleman-Wunsch	46-50	5	NA	0.17 (0.6)
			4	NA	0.18 (0.6)
			3	NA	0.19 (0.6)
			2	NA	0.22 (0.7)
			1	NA	0.19 (0.6)
V9	blastn	10-11	5	2	0.26 (1.5)
			4	2	0.26 (1.5)
			3	2	0.27 (1.5)
			2	2	0.26 (1.4)
			1	2	0.26 (1.4)
			4	1	0.23 (1.4)
			3	1	0.24 (1.3)
			2	1	0.24 (1.3)
	Gotoh	52-55	5	2	0.12 (0.8)
			4	2	0.13 (0.8)
			3	2	0.15 (0.8)
			2	2	0.16 (0.8)
			1	2	0.18 (0.8)
			4	1	0.13 (0.8)
			3	1	0.16 (0.8)
			2	1	0.16 (0.8)
			1	1	0.18 (0.8)
	Needleman-Wunsch	64-65	5	NA	0.15 (0.8)
			4	NA	0.17 (0.8)
			3	NA	0.17 (0.8)
			2	NA	0.19 (0.9)
			1	NA	0.17 (0.8)

a The average percentage difference in similarity between the template sequence and the SILVA aligned candidate sequence and the difference in similarity between the template sequence and the candidate sequence aligned by the different implementations. Positive values indicate the candidate alignment is more similar to the template sequence and negative values are less similar. Values in parentheses indicate the standard deviation.
