# Supplementary material for: A High-Throughput DNA Sequence Aligner for Microbial Ecology Studies
Source: PLoS One. 2009 Dec 14;4(12):e8230. doi: 10.1371/journal.pone.0008230 (PMC2788221; doi:10.1371/journal.pone.0008230)
Supplement: Table S4 — Summary of alignment improvement for various regions extracted from candidate sequences using the blastn, Gotoh, or Needleman-Wunsch pairwise alignment algorithms when the best region-specific template was selected for each candidate sequence. (0.10 MB PDF) [file pone.0008230.s004.pdf]

Table S4. Summary of alignment improvement for various regions extracted from candidate sequences using the blastn, Gotoh, or Needleman-Wunsch pairwise alignment algorithms when the best region-specific template was selected for each candidate sequence.

Region	Alignment method	Speed (seq/s)	Gap opening	Gap extension	% Δsimilarity (sd)[a]
V14	blastn	10	5	2	0.42 (1.1)
			4	2	0.41 (1.1)
			3	2	0.42 (1.1)
			2	2	0.40 (1.0)
			1	2	0.42 (1.0)
			4	1	0.31 (0.9)
			3	1	0.34 (0.8)
			2	1	0.37 (0.8)
	Gotoh	31	5	2	0.22 (0.5)
			4	2	0.23 (0.5)
			3	2	0.26 (0.6)
			2	2	0.28 (0.6)
			1	2	0.35 (0.7)
			4	1	0.24 (0.5)
			3	1	0.28 (0.6)
			2	1	0.32 (0.6)
			1	1	0.43 (0.7)
	Needleman-Wunsch	32-39	5	NA	0.26 (0.6)
			4	NA	0.30 (0.6)
			3	NA	0.35 (0.7)
			2	NA	0.44 (0.7)
			1	NA	0.41 (0.7)
V12	blastn	11	5	2	0.63 (1.9)
			4	2	0.62 (1.9)
			3	2	0.64 (1.8)
			2	2	0.63 (1.7)
			1	2	0.66 (1.6)
			4	1	0.50 (1.5)
			3	1	0.57 (1.5)
			2	1	0.61 (1.5)
	Gotoh	53-55	5	2	0.36 (1.0)
			4	2	0.37 (1.0)
			3	2	0.43 (1.0)
			2	2	0.46 (1.1)
			1	2	0.56 (1.2)
			4	1	0.38 (1.0)
			3	1	0.46 (1.1)
			2	1	0.51 (1.1)
			1	1	0.65 (1.2)
	Needleman-Wunsch	51-58	5	NA	0.43 (1.1)
			4	NA	0.49 (1.1)
			3	NA	0.55 (1.2)
			2	NA	0.66 (1.2)
			1	NA	0.64 (1.2)
V2	blastn	10-12	5	2	0.29 (1.3)
			4	2	0.29 (1.2)
			3	2	0.29 (1.1)
			2	2	0.29 (1.0)
			1	2	0.32 (1.0)
			4	1	0.25 (0.9)
			3	1	0.28 (1.0)
			2	1	0.30 (1.0)
	Gotoh	76-77	5	2	0.14 (0.7)
			4	2	0.15 (0.7)
			3	2	0.16 (0.7)
			2	2	0.18 (0.7)
			1	2	0.22 (0.8)
			4	1	0.15 (0.7)
			3	1	0.18 (0.7)

			2	1	0.21 (0.8)
			1	1	0.29 (0.8)
	Needleman-Wunsch	77-82	5	NA	0.17 (0.7)
			4	NA	0.19 (0.7)
			3	NA	0.22 (0.8)
			2	NA	0.30 (0.8)
			1	NA	0.30 (0.9)
V23	blastn	10-11	5	2	0.29 (1.0)
			4	2	0.28 (1.0)
			3	2	0.28 (0.9)
			2	2	0.28 (0.9)
			1	2	0.31 (0.8)
			4	1	0.23 (0.7)
			3	1	0.25 (0.7)
			2	1	0.28 (0.7)
	Gotoh	44-47	5	2	0.16 (0.5)
			4	2	0.16 (0.5)
			3	2	0.18 (0.5)
			2	2	0.19 (0.5)
			1	2	0.24 (0.6)
			4	1	0.16 (0.5)
			3	1	0.19 (0.5)
			2	1	0.22 (0.6)
			1	1	0.30 (0.6)
	Needleman-Wunsch	44-50	5	NA	0.18 (0.6)
			4	NA	0.20 (0.6)
			3	NA	0.23 (0.6)
			2	NA	0.31 (0.7)
			1	NA	0.28 (0.7)
V3	blastn	10-12	5	2	0.21 (1.3)
			4	2	0.21 (1.3)
			3	2	0.22 (1.3)
			2	2	0.21 (1.2)
			1	2	0.24 (1.2)
			4	1	0.16 (1.0)
			3	1	0.17 (0.9)
			2	1	0.19 (0.9)
	Gotoh	67-68	5	2	0.11 (0.5)
			4	2	0.11 (0.5)
			3	2	0.13 (0.6)
			2	2	0.13 (0.6)
			1	2	0.17 (0.5)
			4	1	0.12 (0.6)
			3	1	0.13 (0.6)
			2	1	0.16 (0.6)
			1	1	0.21 (0.7)
	Needleman-Wunsch	66-69	5	NA	0.12 (0.6)
			4	NA	0.13 (0.6)
			3	NA	0.16 (0.6)
			2	NA	0.21 (0.7)
			1	NA	0.17 (0.7)
V4	blastn	10-11	5	2	0.10 (0.5)
			4	2	0.10 (0.5)
			3	2	0.10 (0.5)
			2	2	0.10 (0.5)
			1	2	0.11 (0.5)
			4	1	0.09 (0.5)
			3	1	0.10 (0.5)
			2	1	0.11 (0.5)
	Gotoh	85-88	5	2	0.04 (0.3)
			4	2	0.04 (0.3)
			3	2	0.05 (0.3)
			2	2	0.05 (0.3)
			1	2	0.07 (0.3)
			4	1	0.04 (0.3)
			3	1	0.05 (0.3)

			2	1	0.06 (0.3)
			1	1	0.12 (0.4)
	Needleman-Wunsch	90-93	5	NA	0.05 (0.3)
			4	NA	0.05 (0.3)
			3	NA	0.07 (0.3)
			2	NA	0.12 (0.4)
			1	NA	0.10 (0.4)
V6	blastn	10-12	5	2	1.50 (14)
			4	2	1.50 (13)
			3	2	1.46 (13)
			2	2	1.45 (13)
			1	2	1.45 (13)
			4	1	1.48 (13)
			3	1	1.45 (13)
			2	1	1.46 (13)
	Gotoh	125-131	5	2	0.31 (1.8)
			4	2	0.34 (1.8)
			3	2	0.40 (1.7)
			2	2	0.43 (1.7)
			1	2	0.48 (1.7)
			4	1	0.36 (1.7)
			3	1	0.43 (1.7)
			2	1	0.47 (1.7)
			1	1	0.61 (1.9)
	Needleman-Wunsch	128-132	5	NA	0.39 (1.8)
			4	NA	0.43 (1.7)
			3	NA	0.48 (1.7)
			2	NA	0.60 (1.9)
			1	NA	0.64 (1.9)
V89	blastn	10-11	5	2	0.24 (1.0)
			4	2	0.24 (1.0)
			3	2	0.25 (0.9)
			2	2	0.25 (0.9)
			1	2	0.25 (0.9)
			4	1	0.22 (0.9)
			3	1	0.23 (0.9)
			2	1	0.23 (0.8)
	Gotoh	51-52	5	2	0.15 (0.6)
			4	2	0.15 (0.6)
			3	2	0.17 (0.6)
			2	2	0.17 (0.6)
			1	2	0.19 (0.6)
			4	1	0.15 (0.6)
			3	1	0.17 (0.6)
			2	1	0.18 (0.6)
			1	1	0.21 (0.6)
	Needleman-Wunsch	53-56	5	NA	0.17 (0.6)
			4	NA	0.18 (0.6)
			3	NA	0.19 (0.6)
			2	NA	0.22 (0.7)
			1	NA	0.19 (0.6)
V9	blastn	9-10	5	2	0.26 (1.5)
			4	2	0.26 (1.5)
			3	2	0.27 (1.5)
			2	2	0.26 (1.4)
			1	2	0.26 (1.4)
			4	1	0.23 (1.4)
			3	1	0.24 (1.3)
			2	1	0.24 (1.3)
	Gotoh	78-79	5	2	0.13 (0.8)
			4	2	0.13 (0.8)
			3	2	0.15 (0.8)
			2	2	0.16 (0.8)
			1	2	0.18 (0.8)
			4	1	0.14 (0.8)
			3	1	0.16 (0.8)

			2	1	0.16 (0.8)
			1	1	0.18 (0.8)
	Needleman-Wunsch	80-82	5	NA	0.15 (0.8)
			4	NA	0.17 (0.8)
			3	NA	0.17 (0.8)
			2	NA	0.19 (0.9)
			1	NA	0.17 (0.8)

a The average percentage difference in similarity between the template sequence and the SILVA aligned candidate sequence and the difference in similarity between the template sequence and the candidate sequence aligned by the different implementations. Positive values indicate the candidate alignment is more similar to the template sequence and negative values are less similar. Values in parentheses indicate the standard deviation.
